# STEADFAST: Psychotherapeutic Intervention Improves Postural Strategy of Somatoform Vertigo and Dizziness

**DOI:** 10.1155/2015/456850

**Published:** 2015-12-30

**Authors:** Christoph Best, Regine Tschan, Nikola Stieber, Manfred E. Beutel, Annegret Eckhardt-Henn, Marianne Dieterich

**Affiliations:** ^1^Department of Neurology, Vestibular Research Unit, Philipps-University, Baldingerstrasse, 35043 Marburg, Germany; ^2^Department of Neurology, University Medical Center, Johannes Gutenberg University, Langenbeckstrasse 1, 55101 Mainz, Germany; ^3^Department of Psychosomatic Medicine and Psychotherapy, University Medical Center, Johannes Gutenberg University, Langenbeckstrasse 1, 55101 Mainz, Germany; ^4^Department of Psychosomatic Medicine, Bürgerhospital, Tunzhofer Strasse 14-16, 70191 Stuttgart, Germany; ^5^Department of Neurology, Ludwig-Maximilians-University, Marchioninistrasse 15, 81377 Munich, Germany; ^6^German Center for Vertigo and Balance Disorders (IFB) (DSGZ^LMU^), Ludwig-Maximilians-University, Marchioninistrasse 15, 81377 Munich, Germany; ^7^Munich Cluster for Systems Neurology (SyNergy), 81377 Munich, Germany

## Abstract

Patients with somatoform vertigo and dizziness (SVD) disorders often report instability of stance or gait and fear of falling. Posturographic measurements indeed indicated a pathological postural strategy. Our goal was to evaluate the effectiveness of a psychotherapeutic and psychoeducational short-term intervention (PTI) using static posturography and psychometric examination. Seventeen SVD patients took part in the study. The effects of PTI on SVD were evaluated with quantitative static posturography. As primary endpoint a quotient characterizing the relation between horizontal and vertical sway was calculated (*Q*_*H*/*V*_), reflecting the individual postural strategy. Results of static posturography were compared to those of age- and gender-matched healthy volunteers (*n* = 28); baseline measurements were compared to results after PTI. The secondary endpoint was the participation-limiting consequences of SVD as measured by the Vertigo Handicap Questionnaire (VHQ). Compared to the healthy volunteers, the patients with SVD showed a postural strategy characterized by stiffening-up that resulted in a significantly reduced body sway quotient before PTI (patients: *Q*_*H*/*V*_ = 0.31 versus controls: *Q*_*H*/*V*_ = 0.38; *p* = 0.022). After PTI the postural behavior normalized, and psychological distress was reduced. PTI therefore appears to modify pathological balance behaviour. The postural strategy of patients with SVD possibly results from anxious anticipatory cocontraction of the antigravity muscles.

## 1. Introduction

Nonorganic vertigo disorders are the focus of an ongoing debate on various concepts, definitions, and diagnostic criteria. Well established concepts are represented by phobic postural vertigo, space and motion phobia, and visual vertigo [1–3]. Due to various underlying causal psychopathological mechanisms (e.g., phobia, anxiety, and depressive disorders; cognitive-behavioral and psychological mechanisms, role of attention, and perception) [4], patients with nonorganic vertigo disorders present with a broad variety of signs and symptoms. A correlation of vestibular abnormalities on vestibular examination batteries on the one hand and psychological strain on the other hand was discussed contradictorily [5]. Despite treatment, many of these patients (86%) have high levels of participation limitations and psychological distress in their daily life [6]. Johansson and coworkers performed a cognitive behavioural therapy in a group of rather unselected patients with recurrent vertigo, including somatoform disorders. Patients experienced an improvement of walking and of dizziness associated handicap, notwithstanding that anxiety and depression remained unchanged [7]. In a comparable design Andersson and colleagues presented an improvement of dizziness associated handicap and anxiety symptoms in another unselected group of vertigo patients by means of cognitive behavioural therapy [8]. Finally Holmberg and colleagues in an open controlled trial initially found positive treatment effects of cognitive behavioural therapy in patients with phobic postural vertigo [9]. After a follow-up period of one year, these positive treatment effects were no longer present [10]. In conclusion, a standardized diagnostic classification and a standardized effective treatment with lasting relief of symptoms for somatoform vertigo and dizziness (SVD) patients are lacking.

To improve the diagnostic and therapeutic management of these patients, we followed a standardized diagnostic classification according to the Diagnostic and Statistical Manual of Mental Disorders-IV (DSM IV) and the International Classification of Diseases-10 (ICD-10) criteria, in order to identify patients with “somatoform vertigo disorder” (SVD) and to develop a standardized psychotherapeutic intervention (PTI) in a pilot study [11]. The aim of the current study was to evaluate the effects of PTI on postural behaviour and psychological distress. We hypothesized that the primary endpoint is indicated by (i) a stiffening-up strategy of postural behavior before treatment and a secondary endpoint is signalled (ii) by a positive modulation of postural control and relief from the psychological distress after PTI.

## 2. Materials and Methods

### 2.1. Patients

In this prospective study 17 SVD patients were examined and compared to 28 age- and gender-matched healthy controls. The mean age of the patients was 52.9 ± 14.1 years (8 females; 9 males) and that of the healthy controls was 50.2 ± 13.2 years (13 females; 15 males). Staff members of the Department of Neurology as well as relatives of the included patients were recruited as healthy controls. Before entering the study, all healthy participants underwent the same vestibular testing as did the included patients (for details, see the following).

Twelve patients had a primary somatoform vertigo disorder (etiology: *n* = 4 anxiety, *n* = 7 somatoform disorder, and *n* = 1 depression) without any history of a vestibular lesion. Five patients had a secondary somatoform vertigo disorder following a vestibular disorder. Of the 17 initially included patients, three did not complete the intervention and were lost to follow-up. Of the 14 patients receiving the intervention, one patient refused to participate in the posturographic measurement after completing the intervention. Therefore, 13 patients finished the whole program. Patients as well as healthy volunteers underwent a detailed clinical neurological examination, a neurophysiological vestibular test battery, and psychosomatic diagnostic procedures. Patients were diagnosed to have SVD if they fulfilled the diagnostic criteria described below.

### 2.2. Diagnostic Criteria of Somatoform Vertigo and Dizziness Disorders

(1) It is subjectively perceived recurrent or persisting vertigo or dizziness, disturbances of stance and gait, or spatial orientation.

(2) Normal findings on neurotological examinations: signs of an earlier vestibular disorder already compensated for were categorized as nonpathological.

(3) It is failure to fulfil the diagnostic criteria of organic vestibular vertigo syndromes.

(4) There are positive criteria according to the Diagnostic and Statistical Manual of Mental Disorders-IV (DSM IV) and the International Classification of Diseases-10 (ICD-10), thus confirming the presence of a somatoform disorder.

### 2.3. Neurological and Vestibular Examination

All participants (patients as well as healthy subjects) underwent a neurological examination including positioning manoeuvres, stepping and head impulse test, examination with Frenzel's glasses, head-shaking test, and a clinical screening for central ocular motor and vestibular deficits. A neurophysiological vestibular examination was performed using fundus photographs and adjustments of the subjective visual vertical (SVV) to detect otolith dysfunction. A binocular 3D video-oculography (VOG) including an ocular motor part and screening for semicircular canal paresis by rotatory and caloric testing were also carried out. Finally, cervical vestibular evoked myogenic potentials (cVEMP) were performed to detect saccular dysfunction.

### 2.4. Static Posturography

The patient's postural control was evaluated using a stabilometer platform (Kistler Type 9286A, Winterthur, Switzerland; for detailed posturographic procedures see [12]). Body sway was measured by sway path values (SP) of the center of foot pressure (COP). Sway paths were calculated for anterior-posterior (*x*-axis) and lateral directions (*y*-axis). The change in transduced force in the vertical direction was calculated and also expressed as a sway path (*z*-axis). Furthermore, the root mean square (RMS) values were analyzed in the *x*-, *y*-, and *z*-axes. Finally, a fast Fourier transformation (FFT) analysis was performed to quantify the body sway activity depending on the frequency spectrum (for details see [12]). Patients were examined at baseline and after the intervention. The results for the 17 included patients at baseline were compared to those of the healthy controls (*n* = 28). Only 13 patients participated in the complete psychotherapeutic intervention and in all investigations. The posturographic data of these patients before the intervention were compared with their results after the intervention. Measurements were performed in ten different conditions and analysed offline after data offset elimination and body weight adapted normalization.

Conditions are as follows: (1) eyes open (EO), (2) eyes closed (EC), (3) eyes open, head reclined 45° (EO-Reclin), and (4) eyes closed, head reclined 45° (EC-Reclin). A reclination of the head was performed by a dorsal flexion, so that the participating patients and volunteers looked upwards, while standing still in the experimental setup. These initial four conditions were repeated after inserting a layer of rubber foam underneath the rigid platform. The following measurements were then made.

Conditions are as follows: (5) eyes open on rubber-foam layer (EO-Foam), (6) eyes closed on rubber-foam layer (EC-Foam), (7) eyes open, head reclined 45° on rubber-foam layer (EO-Reclin-Foam), and (8) eyes closed, head reclined 45° on rubber-foam layer (EC-Reclin-Foam).

For conditions (9) and (10) patients performed tandem stance, their eyes open for condition (9) (EO-Tandem-Foam) and closed for condition (10) (EC-Tandem-Foam). Conditions were recorded for 30 seconds (sampling rate: 200 Hz). If patients could not maintain their balance, this was considered “stepping out of the setting,” that is, “interruption.”

A quotient of horizontal and vertical sway was calculated: the cumulative sway path in the horizontal plane (sum vector of *x*- and *y*-sway) was divided by the vertical sway (quotient of horizontal/vertical sway = *Q*_*H*/*V*_). This quotient was used as primary endpoint.

### 2.5. Psychometric Examination

Patients were examined for mental disorders using the SCID (Structural Clinical Interview for DSM-IV Axis I) [13]. For psychometric testing the Vertigo Handicap Questionnaire (VHQ) was used [14]. The VHQ assesses the handicapping consequences of symptoms related to vertigo and dizziness, including disease-specific restrictions of physical activities, social life, and leisure activities.

### 2.6. Psychotherapeutic Intervention

The PTI program was implemented in a group setting of maximally eight patients (one group with six and one with eight patients). Therapy was on an outpatient basis; appointments were once a week, PTI lasted for 90 minutes, and ten sessions were completed within 3 months. The intervention had five components: (1)* psychoeducation*: it is transfer of knowledge of underlying reasons and mechanisms for continuing symptoms, (2)* health care utilization*: frequent consultations of physicians and diagnostics, such as repetitive MRI scans, were analyzed (patients were informed about the diagnostic and therapeutic procedures), (3)* training*: balance training was performed on a physical and mental basis; handling and realization of body integrity and focus of attention on vertiginous sensations were analyzed, (4)* exposition*: vertigo and dizziness-inducing situations were identified; patients were taught to expose themselves to such situations and were given a “rescue strategy” in case of overwhelming vertigo and dizziness during the process, and (5)* subjective well-being*: quality of life was determined by analyzing resources and competences of the individual patients, and their coping strategies were appropriately modified.

### 2.7. Statistical Analysis

Differences for SP, RMS of SP, and sway activity (FFT) between the patients and healthy controls were assessed using a univariate ANOVA after a normal distribution of data was confirmed. Differences before and after PTI were analyzed by repeated measures ANOVA. The* primary endpoint* of the analyses was the horizontal sway/vertical sway ratio *Q*_*H*/*V*_. The* secondary endpoint* of the analyses was the change in handicap as indicated by the VHQ. Further results of the examinations were also reported. Due to the exploratory character of the study alpha-adjustment of the additional parameters was not performed. All computations were carried out using IBM SPSS Statistics, version 20. The alpha error was set at 5%.

### 2.8. Ethical Considerations

Patients gave their written informed consent; the study was approved by the local ethics committee and was performed in accordance with the Declaration of Helsinki.

## 3. Results

### 3.1. Neurological and Vestibular Examination

None of the patients or the healthy controls exhibited signs of an acute vestibular or central ocular motor dysfunction. Only four of the patients showed signs of an earlier vestibular neuritis with persisting incomplete unilateral canal paresis, but no signs of ongoing vestibular pathology, for example, no spontaneous nystagmus, head-shaking nystagmus, normal head impulse test, no pathological ocular torsion, pathological tilts of SVV, and normal cVEMPs.

### 3.2. Static Posturography

#### 3.2.1. Baseline: Comparison of Patients and Healthy Controls

**Figure 1:**
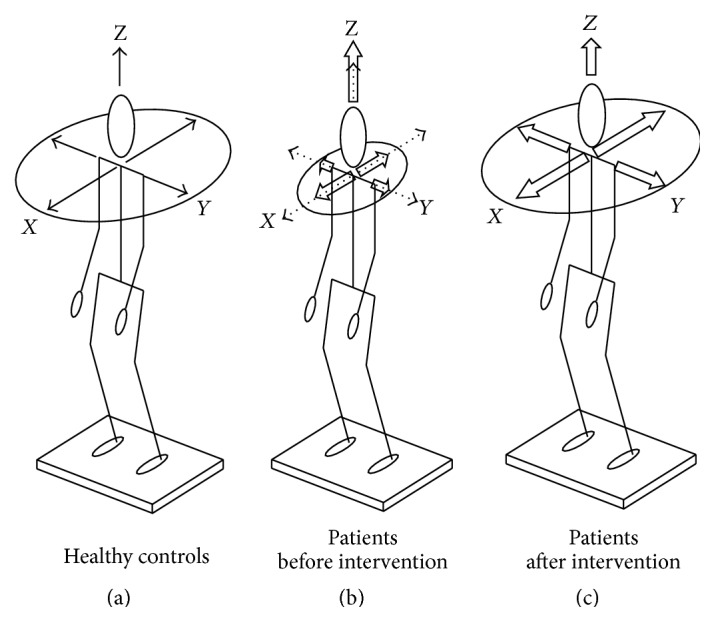
Schematic illustration of postural control. (a) Healthy controls; black vectors in *x*-, *y*-, and *z*-directions indicate physiological body sway activity; (b) SVD patients at baseline; the dotted vectors indicate normal values (healthy controls) and white vectors pathological body sway with reduced horizontal sway and increased vertical sway of the SVD patients. (c) Improvement of postural control after the therapy.

**Figure 2:**
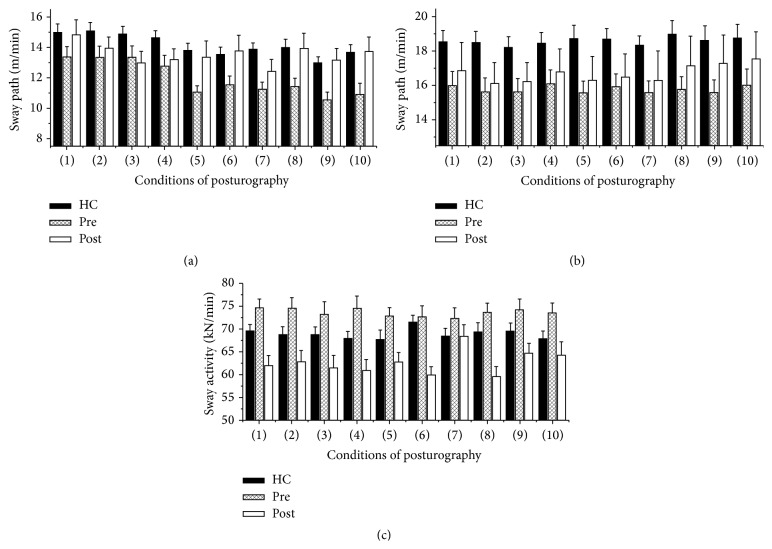
Illustration of absolute and cumulative sway path values in meter/minute (m/min) over the time course of each examination condition. The black columns represent the healthy controls, the hashed columns the patients before PTI, and the white columns the patients after PTI ((a) *x*-axis in m/min, (b) *y*-axis in m/min, and (c) *z*-axis in kN/min). The various conditions of posturographic measurements are displayed along the *x*-axis of (a–c) (conditions (1)–(10)).

Analysis of the sway ratio *Q*_*H*/*V*_ as* primary endpoint*: patients showed a reduced quotient of 0.31 ± 0.01 in comparison to 0.38 ± 0.01 in the controls, thus expressing an elevated vertical sway (*F* = 5.503, *p* = 0.022). A test of the isolated directions and planes revealed that patients had a significantly reduced body sway in the horizontal plane in comparison to that of the healthy subjects (*x*-/*y*-axis: Table 1; Figures 1(a), 1(b), 2(a), and 2(b)). Body sway along the vertical direction was elevated in the SVD patients (*z*-axis: Table 1; Figures 1(a), 1(b), and 2(c)).

While the root mean square values for the *x*- and *y*-axes did not show relevant differences, these values were significantly elevated for the *z*-axis in the patients (Table 1). Patients had a mean of 4.2 interruptions over the 10 conditions, while only 0.6 interruptions were recorded for the healthy controls (*p* < 0.001). FFT analyses revealed a significantly elevated activity in the low frequency (0.1–2.4 Hz), medium frequency range (2.5–3.5 Hz), and high frequency range (3.5–8 Hz) for the patients. At higher frequencies (11–18 Hz) patients had significantly decreased body sway activity (Table 2).

#### 3.2.2. After the Psychotherapeutic Intervention

After the intervention, the postural control of the patients normalized. Body sway in the horizontal plane for conditions 1–10 was elevated in comparison to baseline measurements (*x*-/*y*-axis: Figures 1(c), 2(a)–2(c), and Table 3). There was a reduction of body sway along the vertical *z*-axis (Figure 1(c), Table 3).

In parallel, the quotient *Q*_*H*/*V*_ also showed a tendency towards normalization (*p* = 0.054, *F* = 4.164). However, the number of interruptions did not improve significantly (*p* = 0.453). FFT analyses showed a change in body sway activity only for the higher frequency range (11–18 Hz) and only along the *z*-axis. After the intervention, sway activity along the *z*-axis was reduced under all conditions (*p* = 0.029, *p* = 0.036, Figure 2(c)).

### 3.3. Psychometric Testing

Analysis of the improved consequences of vertigo in daily life activities as indicated by the* secondary endpoint* revealed a reduction of psychological distress after the intervention (baseline score = 31.1 ± 5.2 (SD), postintervention score = 29.3 ± 8.9 (SD)). However, this improvement did not reach the level of significance (*p* = 0.142).

## 4. Discussion

To the best of our knowledge, this is the first study to demonstrate that the postural behaviour in an unselected group of patients with SVD improved after a psychotherapeutic intervention. At baseline in comparison to healthy controls the patients showed significant stiffening-up in their postural strategy with reduced horizontal and increased vertical body sway. This could be demonstrated by the primary endpoint parameter of the sway ratio *Q*_*H*/*V*_ but also by the single comparisons of all sway directions under the 10 conditions of the static posturography. This pathological stiffening-up strategy had then been modulated towards a healthy postural behaviour after the intervention. In parallel, the handicapping psychological consequences of the subjectively perceived vertigo and dizziness were also reduced, even though this failed to become significant. Conclusions and interpretation of these results are, however, limited by the fact that the pilot study design did not include a randomized control group, and the number of patients was also small.

Nonetheless, the standardized diagnostic classification of somatoform vertigo disorders on the basis of sound international guidelines displays one strength of the study. In addition to the standardized psychometric procedures, patients as well as healthy volunteers underwent a very detailed neurological and vestibular examination. On the one hand, this approach resulted in an unselected group of patients with somatoform vertigo disorders and, on the other hand, in a healthy control group without any latent psychological comorbidity or any neurological or vestibular disorders. Finally, the combination of a psychotherapeutic intervention with the objective and quantitative measurement of postural behaviour enabled us to detect reliable interactions between psychometric and neurophysiological parameters.

### 4.1. Cognitive Behavioural Treatment for Vertigo and Dizziness

Up to now, data on the effectiveness of a standardized treatment regimen for patients with SVD have been sparse. This is especially true when comparing neurophysiological parameters before and after psychotherapeutic interventions. In a population of selected patients with phobic postural vertigo Holmberg and coworkers showed that various psychometric tools can measure the significant improvement of vertigo-induced psychological strain due to cognitive- behavioral therapy [9]. The treatment effect, however, wore off after 1 year [10]. In an unselected population of elderly dizzy patients Johansson and coworkers proved the positive effects of cognitive-behavioral therapy, and Andersson and coworkers also found similar effectiveness in a younger population [7, 8]. None of these studies reported neurotological measures of these treatment effects. In agreement with these controlled and randomized studies, our current investigation also pointed to an improvement of vertigo-induced psychological distress and handicap. However, in addition to a psychometric assessment, we report on an objective improvement of involuntary postural behaviour.

### 4.2. Effects after PTI as Measured by Static Posturography

Previous studies proved that a rehabilitation program in patients with organic vestibular disorders led to a reduction of the initially elevated horizontal body sway [15, 16]. Our patients exhibited the opposite effect: the pathologically reduced horizontal body sway was increased but normalized after the psychotherapeutic intervention. Therefore, unspecific effects, such as training effects of physiotherapy and vestibular rehabilitation, seem unlikely. Moreover, data from our own laboratory revealed no significant difference of posturographic strategy in healthy controls when repeatedly tested. Effects of multiple or serial testing can therefore be ruled out (unpublished data). The improvement of the postural behaviour after the PTI confirms our earlier conclusions that the abnormalities in SVD patients are not the consequence of an organic disorder or dysfunction [17] but instead reflect an involuntary change of postural strategies. These results contradict earlier interpretations that anxiety disorders are caused by a vestibular disorder/dysfunction [18–21]. In contrast, primary and secondary SVD may cause postural abnormalities due to involuntary balance strategies, but they can be normalized with adequate therapy.

### 4.3. Interpretation of Posturographic Parameters

In a former study, Querner and coworkers performed a posturographic examination in patients with phobic postural vertigo (PPV) compared to healthy controls [22]. As main outcome parameter the body sway was calculated and differentiated into 4 groups of frequency range as expression of frequency dependent body sway activity. PPV patients performed significantly poorer as compared to healthy controls throughout the whole frequency range from 0.1 Hz to 19 Hz. The effect of increased sum body sway activity was pronounced in the medium frequency range 3.53 Hz–8 Hz; however, it was also present in all the other frequency ranges. Within the high frequency range, patients in this study showed better performance in one of the conditions as compared to healthy controls. In our current study, the frequency ranges (a) 0.1 Hz–2.4 Hz, (b) 2.4 Hz–3.5 Hz, and (c) 3.5 Hz–8 Hz showed a very comparable pattern with increased body sway activity. Within the high frequency range from 11 Hz to 19 Hz, the patients presented with a reduced body sway activity. This effect may adhere to results demonstrated by Querner and coworkers. In conclusion, our current data nicely fit with previous examinations in nonorganic vertigo patients expressing increased body sway activity as analysed by FFT. Furthermore, PPV patients showed particular poor performance on easy balance task with an improvement of their performance on more difficult tasks. Comparable with this finding, in the current study SVD patients showed elevated body sway for conditions (1)–(4) with a remarkable improvement at more difficult conditions (5)–(10) (Figure 2). Finally, the RMS of vertical body sway along the *z*-axis was significantly elevated for every single condition. In conclusion, the various parameters together reflect the pathological postural strategy: (a) the absolute body sway as expressed by sway path was decreased in the horizontal plane while it was elevated in the vertical direction, (b) the RMS along the *z*-axis was also elevated, reflecting the anticipatory stiffening-up, and (c) there is increased body sway activity in almost every frequency range, while the absolute sway was horizontally reduced.

### 4.4. Postural Control Strategy in Patients with Somatoform Vertigo and Dizziness

We found evidence that the pathological strategies of postural control in somatoform vertigo patients result from decreased horizontal and elevated vertical body sway. These findings corroborate posturographic studies in patients with phobic postural vertigo [23, 24] showing reduced horizontal body sway during visual stimulation and an improvement of balance performance with increasing difficulty of the balance tasks. The pathological pattern was comparable to the strategy of healthy subjects when they maintain balance during a demanding balance task (e.g., slippery ground). In patients with somatoform vertigo the pathological pattern can be attributed to anxious anticipation: with anxious control of body posture, the preinnervation of the muscles (cocontraction) during the intended movement inhibits and modifies the movement. Subsequently the reafference signal is inappropriate and results in a sensorimotor mismatch [1, 25]. Thus, increased self-monitoring and introception seem to be the likely mechanisms in patients with somatoform vertigo and dizziness. In our study PTI improved the patient's understanding of balance performance and illness perception so that they learned to normalize their postural control by relinquishing the stiffening-up strategy.

## 5. Conclusion

Psychoeducational interventions led to a normalization of postural behaviour in our patient group. In parallel, the vertigo/dizziness-induced psychological distress and handicapping consequences decreased. We conclude that while PTI is able to modify the pathological balance behaviour, it cannot sufficiently modify the underlying psychopathology. Since the patients profited from the altered postural behaviour and the psychological relief, the intervention should be applied as early as possible. After the short-term intervention patients with somatoform vertigo and dizziness disorders then need to apply further psychotherapeutic modalities to achieve long-lasting therapeutic effects and be sufficiently freed from the underlying psychopathology.

## Figures and Tables

**Table 1:** Comparison of postural control in SVD patients and in healthy subjects. Significance levels as displayed by *p* values for all different posturographic conditions (1)–(10) and for all evaluated parameters (sway path = SP, root mean square of sway path = RMS) of the patients' postural control at baseline in comparison to that of healthy controls. The numbers represent the *p* values of the comparisons. Effect sizes are displayed by *F*-values. Significantly reduced body sway was found for the horizontal plane as well as increased body sway for the vertical direction. In this context the term sway with an upward arrow indicates elevated sway values of the patients in comparison to the normal subjects; downward arrows indicate reduced sway values of the patients.

Univariate ANOVA							
Patients with somatoform vertigo (*n* = 17) versus healthy controls (*n* = 28)							
	SP(*X*)	SP(*Y*)	SP(*XY*)	SP(*Z*)	RMS(*X*)	RMS(*Y*)	RMS(*Z*)
EO		*Sway* ↓	*Sway* ↓	*Sway* ↑			*Sway* ↑
	ns	*F* = 5.128	*F* = 4.681	*F* = 4.412	ns	ns	*F* = 11.565
		*p* = 0.028	*p* = 0.036	*p* = 0.041			*p* = 0.001

EC		*Sway* ↓	*Sway* ↓	*Sway* ↑			*Sway* ↑
	ns	*F* = 6.745	*F* = 5.438	*F* = 4.408	ns	ns	*F* = 11.553
		*p* = 0.012	*p* = 0.024	*p* = 0.050			*p* = 0.001

EO-Reclin		*Sway* ↓	*Sway* ↓				*Sway* ↑
	ns	*F* = 6.095	*F* = 5.046	ns	ns	ns	*F* = 11.486
		*p* = 0.017	*p* = 0.029				*p* = 0.001

EC-Reclin	*Sway* ↓	*Sway* ↓	*Sway* ↓	*Sway* ↑			*Sway* ↑
	*F* = 5.009	*F* = 5.052	*F* = 5.517	*F* = 5.749	ns	ns	*F* = 11.553
	*p* = 0.03	*p* = 0.029	*p* = 0.023	*p* = 0.02			*p* = 0.001

EO-Foam							*Sway* ↑
	ns	ns	ns	ns	ns	ns	*F* = 9.376
							*p* = 0.001

EC-Foam	*Sway* ↓	*Sway* ↓	*Sway* ↓		*Sway* ↑		*Sway* ↑
	*F* = 6.076	*F* = 7.088	*F* = 7.101	ns	*F* = 6.492	ns	*F* = 11.625
	*p* = 0.017	*p* = 0.011	*p* = 0.001		*p* = 0.014		*p* = 0.001

EO-Reclin-Foam	*Sway* ↓	*Sway* ↓	*Sway* ↓				*Sway* ↑
	*F* = 14.47	*F* = 9.056	*F* = 11.689	ns	ns	ns	*F* = 11.520
	*p* = 0.000	*p* = 0.004	*p* = 0.001				*p* = 0.001

EC-Reclin-Foam	*Sway* ↓	*Sway* ↓	*Sway* ↓		*Sway* ↑		*Sway* ↑
	*F* = 8.237	*F* = 6.669	*F* = 7.806	ns	*F* = 4.087	ns	*F* = 11.246
	*p* = 0.006	*p* = 0.013	*p* = 0.007		*p* = 0.049		*p* = 0.002

EO-Tandem-Foam	*Sway* ↓	*Sway* ↓	*Sway* ↓				*Sway* ↑
	*F* = 12.10	*F* = 5.250	*F* = 7.536	ns	ns	ns	*F* = 11.503
	*p* = 0.001	*p* = 0.026	*p* = 0.008				*p* = 0.001

EC-Tandem-Foam	*Sway* ↓	*Sway* ↓	*Sway* ↓	*Sway* ↑			*Sway* ↑
	*F* = 9.358	*F* = 4.371	*F* = 6.340	*F* = 4.351	ns	ns	*F* = 4.073
	*p* = 0.004	*p* = 0.042	*p* = 0.015	*p* = 0.042			*p* = 0.049

**Table 2:** Comparison of body sway activity in SVD patients and healthy subjects by fast Fourier transformation (FFT). Results: *p* values of sway activity analyzed by a fast Fourier transform (FFT) in different frequency ranges. For characterization of effect sizes, the *F*-values are also displayed. Significantly elevated body sway for the horizontal plane and the vertical direction was observed for frequencies up to 8 Hz; a significantly reduced body sway activity was observed in the high frequency range.

Univariate ANOVA						
Patients with somatoform vertigo (*n* = 17) versus healthy controls (*n* = 28)						
	FFT Integral 0.1–2.4 Hz			FFT Integral 2.4–3.5 Hz		
	*X*	*Y*	*Z*	*X*	*Y*	*Z*

EO				*Sway* ↑		
	ns	ns	ns	*F* = 5.509	ns	ns
				*p* = 0.027		
EC	*Sway* ↑	*Sway* ↑		*Sway* ↑		
	*F* = 6.156	*F* = 10.399	ns	*F* = 5.782	ns	ns
	*p* = 0.003	*p* = 0.001		*p* = 0.014		
EO-Reclin	*Sway* ↑	*Sway* ↑				
	*F* = 5.551	*F* = 6.695	ns	ns	ns	ns
	*p* = 0.029	*p* = 0.011				
EC-Reclin	*Sway* ↑	*Sway* ↑		*Sway* ↑		
	*F* = 5.559	*F* = 5.071	ns	*F* = 4.873	ns	ns
	*p* = 0.006	*p* = 0.024		*p* = 0.027		
EO-Foam						
	ns	ns	ns	ns	ns	ns
					
EC-Foam	*Sway* ↑	*Sway* ↑	*Sway* ↑			
	*F* = 12.228	*F* = 13.125	*F* = 5.568	ns	ns	ns
	*p* = 0.001	*p* = 0.001	*p* = 0.026			
EO-Reclin-Foam						
	ns	ns	ns	ns	ns	ns
					
EC-Relin-Foam		*Sway* ↑				
	ns	*F* = 7.543	ns	ns	ns	ns
		*p* = 0.011				
EO-Tandem-Foam		*Sway* ↑				
	ns	*F* = 6.540	ns	ns	ns	ns
		*p* = 0.013				
EC-Tandem-Foam					*Sway* ↑	
	ns	ns	ns	ns	*F* = 6.123	ns
					*p* = 0.014	

	FFT Integral 3.5–8 Hz			FFT Integral 11–19 Hz		
	*X*	*Y*	*Z*	*X*	*Y*	*Z*

EO					*Sway* ↓	
	ns	ns	ns	ns	*F* = 6.505	ns
					*p* = 0.025	
EC				*Sway* ↓	*Sway* ↓	
	ns	ns	ns	*F* = 5.617	*F* = 6.693	ns
				*p* = 0.032	*p* = 0.009	
EO-Reclin				*Sway* ↓	*Sway* ↓	
	ns	ns	ns	*F* = 4.996	*F* = 7.086	ns
				*p* = 0.046	*p* = 0.015	
EC-Reclin				*Sway* ↓	*Sway* ↓	
	ns	ns	ns	*F* = 10.878	*F* = 7.214	ns
				*p* = 0.005	*p* = 0.014	
EO-Foam	*Sway* ↑				*Sway* ↓	
	*F* = 4.179	ns	ns	ns	*F* = 7.689	ns
	*p* = 0.043				*p* = 0.013	
EC-Foam	*Sway* ↑	*Sway* ↑	*Sway* ↑		*Sway* ↓	
	*F* = 5.825	*F* = 5.131	*F* = 7.122	ns	*F* = 6.340	ns
	*p* = 0.023	*p* = 0.028	*p* = 0.011		*p* = 0.005	
EO-Reclin-Foam			*Sway* ↑	*Sway* ↓	*Sway* ↓	
	ns	ns	*F* = 4.361	*F* = 5.115	*F* = 5.630	ns
			*p* = 0.045	*p* = 0.033	*p* = 0.029	
EC-Relin-Foam			*Sway* ↑			
	ns	ns	*F* = 7.525	ns	ns	ns
			*p* = 0.012			
EO-Tandem-Foam			*Sway* ↑	*Sway* ↓	*Sway* ↓	
	ns	ns	*F* = 6.173	*F* = 8.176	*F* = 7.105	ns
			*p* = 0.020	*p* = 0.002	*p* = 0.011	
EC-Tandem-Foam						
	ns	ns	ns	ns	ns	ns
					

**Table 3:** Comparison of postural control in patients with somatoform vertigo before and after PTI.

Repeated measures ANOVA							
Patients with somatoform vertigo before (*n* = 13) versus after therapy (*n* = 13)							
	SP(*X*)	SP(*Y*)	SP(*XY*)	SP(*Z*)	RMS(*X*)	RMS(*Y*)	RMS(*Z*)
EO	*Sway* ↑			*Sway* ↓	*Sway* ↑		
	*F* = 4.875	ns	ns	*F* = 23.236	*F* = 5.222	ns	ns
	*p* = 0.046			*p* = 0.001	*p* = 0.043		

EC	*Sway* ↑			*Sway* ↓	*Sway* ↑		
	*F* = 6.088	ns	ns	*F* = 5.403	*F* = 12.902	ns	ns
	*p* = 0.028			*p* = 0.040	*p* = 0.004		

EO-Reclin	*Sway* ↑				*Sway* ↑		
	*F* = 6.010	ns	ns	ns	*F* = 9.141	ns	ns
	*p* = 0.029				*p* = 0.012		

EC-Reclin				*Sway* ↓			
	ns	ns	ns	*F* = 7.818	ns	ns	ns
				*p* = 0.017			

EO-Foam				*Sway* ↓			
	ns	ns	ns	*F* = 5.726	ns	ns	ns
				*p* = 0.036			

EC-Foam				*Sway* ↓			
	ns	ns	ns	*F* = 12.931	ns	ns	ns
				*p* = 0.004			

EO-Reclin-Foam	ns	ns	ns	ns	ns	ns	ns

EC-Relin-Foam				*Sway* ↓		*Sway* ↑	
	ns	ns	ns	*F* = 10.973	ns	*F* = 6.527	ns
				*p* = 0.007		*p* = 0.027	

EO-Tandem-Foam	ns	ns	ns	ns	ns	ns	ns

EC-Tandem-Foam				*Sway* ↓			
	ns	ns	ns	*F* = 11.232	ns	ns	ns
				*p* = 0.006			

Significance levels as displayed by *p* values for all different posturographic conditions (1)–(10) and for all evaluated parameters (sway path = SP, root mean square of sway path = RMS) of the patients' postural control at baseline in comparison to measurements after the psychotherapeutic intervention. The numbers represent the *p* values of the comparisons. For demonstration of effect size, the *F*-values are presented. Body sway significantly changed towards a healthy posture pattern. Sway in the horizontal plane was increased (indicated by the term sway with an upward arrow) and sway in the vertical axis was significantly reduced (indicated by the term sway with downward arrows) throughout every single posturographic condition.

## Abbreviations and Symbols

ANOVA: Analysis of variance
COP: Center of foot pressure
DSM: Diagnostic and Statistical Manual of Mental Disorders
FFT: Fast Fourier transformation
PTI: Psychotherapeutic and psychoeducational short-term intervention
RMS: Root mean square
SP: Sway path
SVD: Somatoform vertigo and dizziness
VHQ: Vertigo Handicap Questionnaire.
